# MicroRNA‐129‐1‐3p protects cardiomyocytes from pirarubicin‐induced apoptosis by down‐regulating the GRIN2D‐mediated Ca^2+^ signalling pathway

**DOI:** 10.1111/jcmm.14908

**Published:** 2020-01-19

**Authors:** Qi Li, Meng Qin, Qi Tan, Tengteng Li, Zehui Gu, Peng Huang, Liqun Ren

**Affiliations:** ^1^ Department of Experimental Pharmacology and Toxicology School of Pharmacy Jilin University Changchun China; ^2^ The Third Hospital Affiliated of Jinzhou Medical University Jinzhou China; ^3^ Department of Pathology and Pathophysiology Jinzhou Medical University Jinzhou China

**Keywords:** apoptosis, Ca^2+^ signalling pathway, cardiomyocytes, GRIN2D, miR‐129‐1‐3p, pirarubicin

## Abstract

Pirarubicin (THP), an anthracycline anticancer drug, is a first‐line therapy for various solid tumours and haematologic malignancies. However, THP can cause dose‐dependent cumulative cardiac damage, which limits its therapeutic window. The mechanisms underlying THP cardiotoxicity are not fully understood. We previously showed that MiR‐129‐1‐3p, a potential biomarker of cardiovascular disease, was down‐regulated in a rat model of THP‐induced cardiac injury. In this study, we used Gene Ontology (GO) and Kyoto Encyclopedia of Genes and Genome (KEGG) pathway enrichment analyses to determine the pathways affected by miR‐129‐1‐3p expression. The results linked miR‐129‐1‐3p to the Ca^2+^ signalling pathway. TargetScan database screening identified a tentative miR‐129‐1‐3p‐binding site at the 3′‐UTR of GRIN2D, a subunit of the N‐methyl‐D‐aspartate receptor calcium channel. A luciferase reporter assay confirmed that miR‐129‐1‐3p directly regulates GRIN2D. In H9C2 (rat) and HL‐1 (mouse) cardiomyocytes, THP caused oxidative stress, calcium overload and apoptotic cell death. These THP‐induced changes were ameliorated by miR‐129‐1‐3p overexpression, but exacerbated by miR‐129‐1‐3p knock‐down. In addition, miR‐129‐1‐3p overexpression in cardiomyocytes prevented THP‐induced changes in the expression of proteins that are either key components of Ca^2+^ signalling or important regulators of intracellular calcium trafficking/balance in cardiomyocytes including GRIN2D, CALM1, CaMKⅡδ, RyR2‐pS2814, SERCA2a and NCX1. Together, these bioinformatics and cell‐based experiments indicate that miR‐129‐1‐3p protects against THP‐induced cardiomyocyte apoptosis by down‐regulating the GRIN2D‐mediated Ca^2+^ pathway. Our results reveal a novel mechanism underlying the pathogenesis of THP‐induced cardiotoxicity. The miR‐129‐1‐3p/Ca^2+^ signalling pathway could serve as a target for the development of new cardioprotective agents to control THP‐induced cardiotoxicity.

## 1 INTRODUCTION

Anthracyclines are a fundamental class of antineoplastic drugs that are used to treat more types of cancer than any other form of chemotherapy.^1^ These drugs act by intercalating into DNA and interacting with topoisomerase II, thereby blocking DNA replication, RNA transcription and protein synthesis.^2^ However, anthracycline administration is often accompanied by dose‐dependent and cumulative cardiotoxicity, ranging from transient cardiac dysfunction to congestive heart failure.^3^ According to the 2011 report from the China Society of Clinical Oncology (CSCO),^4^ more than 50% of patients who have received an anthracycline treatment within the past 6 years have developed subclinical changes in left ventricular function and structure as demonstrated by echocardiographic evaluation. Pirarubicin (THP) is a fourth‐generation anthracycline that is less cardiotoxic than the first‐generation anthracyclines, including doxorubicin (DOX),^5^ and is therefore widely used in clinical practice. However, patients who receive THP treatment can still suffer significant cardiac injuries.^6^, ^7^ Thus, novel therapeutic agents that help combat THP‐induced cardiotoxicity are required.

The mechanisms underlying anthracycline‐induced cardiotoxicity are not fully understood. Studies in recent years have demonstrated that anthracyclines trigger excessive mitochondrial reactive oxygen species (ROS) production in cardiomyocytes, subsequently inducing calcium overload, mitochondrial dysfunction, autophagy dysregulation, and eventually apoptotic and autophagic cell death.^8^, ^9^, ^10^, ^11^, ^12^, ^13^, ^14^, ^15^, ^16^ Therefore, inhibiting ROS accumulation and calcium overload may be an effective strategy to control THP‐induced cardiotoxicity.

MicroRNAs (miRNAs) are small non‐coding RNAs that regulate gene expression by interacting with the 3′‐UTR of mRNA molecules. miRNAs have been shown to regulate cardiac physiology and pathology.^17^, ^18^, ^19^ Several miRNAs have been implicated in cardiotoxicity caused by anthracyclines.^20^, ^21^ For example, miR‐21 prevents DOX‐induced cardiomyocyte apoptosis by targeting BTG2,^22^ while miR‐208a mediates DOX‐induced cardiotoxicity through regulating GATA4.^23^ Additional in vitro and in vivo studies have implicated miR‐532‐3p, miR‐34a‐5p and miR‐451 in DOX‐induced cardiotoxicity.^18^, ^19^ We recently performed a microarray analysis on myocardial miRNAs in a rat model of THP‐induced myocardial injury and identified 78 dysregulated miRNAs in the THP group compared with control (*P* < .05, |log2(fold change) | > 0.585), of which 50 were up‐regulated and 28 were down‐regulated.^24^

In this study, we performed Gene Ontology (GO) enrichment analysis of the 28 miRNAs down‐regulated by THP using databases in the public domain (http://www.mirbase.org/, http://www.microrna.gr/LncBase/, https://david.ncifcrf.gov/, and http://www.targetscan.org/vert_71/). The results revealed that miR‐129‐1‐3p, which is a potential biomarker of cardiovascular disease,^25^ is highly conserved across species and potentially targets GRIN2D (also known as NMDAR2D, NR2D or GluN2D). GRIN2D is a subunit of the NMDA (N‐methyl‐D‐aspartate) receptor complex, which forms ligand‐gated ion channels with high calcium permeability.^26^ Considering that calcium overload is a contributing factor in anthracycline‐induced myocardial injury, we speculated that miR‐129‐1‐3p might play a role in THP‐induced cardiotoxicity by regulating GRIN2D and calcium homoeostasis. In this study, we investigated the function of miR‐129‐1‐3p in THP‐induced cardiomyocyte apoptosis and the underlying molecular mechanisms involving GRIN2D‐mediated Ca^2+^ signalling.

## 2 MATERIALS AND METHODS

### 2.1 Reagents and materials

THP was purchased from Dahua Pharmaceutical Co., Ltd. The bicinchoninic acid (BCA) protein, Fluo‐3 AM calcium and the Cell Counting Kit‐8 (CCK‐8) were all purchased from Beyotime Institute of Biotechnology. The DCFH‐DA ROS assay kit and the TUNEL Apoptosis Detection Kit (Alexa Fluor 640) were purchased from Shanghai Yeasen Biotech Co., Ltd.. The Annexin V‐FITC Apoptosis Detection Kit was from BD Biosciences.

### 2.2 Cell culture, transfection and THP treatment

The H9C2 rat cardiomyoblast cell line and the mouse HL‐1 cardiac muscle cell line were obtained from Shanghai Institute of Cell Biology and Otwo Biotech Inc, respectively. The cells were maintained in Dulbecco's Modified Eagle Medium (DMEM; Hyclone) with 10% foetal bovine serum (FBS; Hyclone) and penicillin‐streptomycin. All cells were cultured at 37°C in a humidified atmosphere containing 5% CO_2_.

The miR‐129‐1‐3p mimics, mimics negative control (mimics NC), miR‐129‐1‐3p inhibitor and inhibitor negative control (inhibitor NC) (Table 1) were prepared by GenePharma Technology Co., Ltd. (Jiangsu, China). The miR‐129‐1‐3p mimics and miR‐129‐1‐3p inhibitor were labelled with the FAM green fluorescent dye at the 5′ end. The miRNAs were transfected into H9C2 and HL‐1 cells for 8 hours in serum‐free medium using Lipofectamine 2000 (Beijing TransGen Biotech Co., Ltd.) following the manufacturer's instructions. The serum‐free medium was replaced with fresh normal medium after transfection was completed. The transfection efficacy was calculated as the ratio of the number of positive nuclei to the total number of nuclei.

**Table 1:** Sequences of the miR‐129‐1‐3p mimics, inhibitors and negative controls used in cell transfection

	Sequences (5′‐3′)
rno‐miR‐129‐1‐3p mimics	F: AAGCCCUUACCCCAAAAAG
	R: UUUUGGGGUAAGGGCUUUU
mmu‐miR‐129‐1‐3p mimics	F: AAGCCCUUACCCCAAAAAGUAU
	R: ACUUUUUGGGGUAAGGGCUUUU
mimics NC	F: UUCUCCGAACGUGUCACGUTT
	R: ACGUGACACGUUCGGAGAATT
rno‐miR‐129‐1‐3p inhibitor	CUUUUUGGGGUAAGGGCUU
mmu‐miR‐129‐1‐3p inhibitor	AUACUUUUUGGGGUAAGGGCUU
inhibitor NC	CAGUACUUUUGUGUAGUACAA

Abbreviations: F, forward; mmu, Mouse; NC, negative control; R, reverse; rno, Rat.

### 2.3 Cell viability assay

To test the effects of THP on cell viability, THP working solutions were prepared in DMEM. The cells were seeded in 96‐well plates (5 × 10^4^ cells/well) and cultured for 24 hours. The cells were subsequently treated with THP (0‐10 μmol/L) for 24 hours. Cell viability was evaluated using the CCK‐8 assay kit on a Bio‐Rad POLARstar microplate reader.

### 2.4 Measurement of intracellular ROS levels

Intracellular ROS levels were determined using the DCFH‐DA ROS assay kit. In brief, the cells were plated in six‐well plates at a density of 5 × 10^4^ cells/well and cultured for 24 hours. The cells were subsequently treated with THP (5 μmol/L) for 24 hours. After that, the medium was removed, and 1.5 mL of DCFH‐DA (10 μmol/L) was added. The cells were incubated at 37°C for 30 minutes and subjected to analysis under a BX43 fluorescence microscope (Olympus; Tokyo, Japan) at 100 × magnification.

### 2.5 TUNEL assay

Following treatment, cells were fixed in 4% paraformaldehyde and permeabilized in 0.1% Triton‐X 100. Cell apoptosis was assessed using the TUNEL Apoptosis Detection kit. The cells were counterstained with DAPI and analysed under a BX43 fluorescence microscope at 100× magnification. The apoptosis rate was calculated as the ratio of TUNEL‐positive nuclei to total nuclei.

### 2.6 Flow cytometry

Following treatment, cells were harvested, washed three times in cold phosphate buffered saline (PBS) and double‐strained with Annexin V‐FITC and propidium iodide (PI) according to the manufacturer's instructions. The cells were incubated in the dark at room temperature for 15 minutes. Then, 400 μL binding buffer was added and the cells were subjected to flow cytometric analysis.

### 2.7 Bioinformatics analysis

GO and Kyoto Encyclopedia of Genes and Genomes (KEGG) pathway enrichment analyses were performed using public domain databases (https://david.ncifcrf.gov/). Pathway regulation networks were visualized using Cytoscape software mapping. Potential targets for rat or mouse miR‐129‐1‐3p were identified using TargetScan database screening.

### 2.8 Measurement of intracellular calcium levels

Intracellular calcium levels were measured using the fluorescent Ca^2+^‐sensitive dye Fluo‐3 AM. Briefly, after washing with PBS, the cells were loaded with 5 μmol/L Fluo‐3 AM at 37°C for 30 minutes and examined under a BX43 fluorescence microscope at 100× magnification. Intracellular Ca^2+^ accumulation was evaluated as the ratio of Fluo‐3 AM‐positive nuclei to total nuclei.

### 2.9 Dual‐luciferase reporter assay

The wild‐type 3′‐untranslated region (UTR) of GRIN2D containing putative binding sites for miR‐129‐1‐3p was PCR‐amplified using genomic DNA from HL‐1 cells. The corresponding mutant 3′‐UTR was created by altering the seed regions of the miR‐129‐1‐3p binding sites. The wild‐type and mutant 3′‐UTRs were subcloned into the psiCHECK‐2 luciferase vector downstream of the luciferase gene. Both constructs were verified by DNA sequencing. HL‐1 cells were cotransfected with the miR‐129‐1‐3p mimics or miR‐129‐1‐3p inhibitor and the luciferase plasmid comprising the wild‐type or mutant 3′‐UTR in 24‐well plates. Luciferase activity was determined 48 hours after transfection using the Dual‐Luciferase Reporter Assay System (Promega) and normalized to Renilla activity.

### 2.10 Quantitative real‐time PCR (qRT‐PCR)

Total RNA was extracted using TransZol (Beijing TransGen Biotech). Each RNA sample was reverse transcribed into cDNA using the TransStart Top Green qPCR SuperMix kit (Beijing TransGen Biotech). The levels of GRIN2D mRNA and GAPDH mRNA were determined by qRT‐PCR. Total miRNA was isolated using the SanPrep Column MicroRNA Mini‐Preps Kit (Sangon Biological Engineering Technology & Services Co., Ltd). Each miRNA sample was reverse transcribed into cDNA using the MicroRNA First Strand cDNA Synthesis Kit (Sangon). The levels of miRNA‐129‐1‐3p and U6 snRNA were determined by qRT‐PCR using the MicroRNAs Quantitation PCR Kit (Sangon). The PCR primer sequences are shown in Tables 2 and 3. Relative expression levels of GRIN2D and miRNA‐129‐1‐3p were calculated using the 2^−ΔΔCt^ method and normalized to GAPDH and U6, respectively.

**Table 2:** Sequences of primers used in PCR analysis of H9C2 cells

	Sequences (5′‐3′)
GRIN2D	F: GCTACATGGTGCGATACAAC
	R: ATTGTGGCAGATCCCTGAAA
Bax	F: GATCAGCTCGGGCACTTTA
	R: TGTTTGCTGATGGCAACTTC
Bcl‐2	F: CCGGGAGAACAGGGTATGATAA
	R: CCCACTCGTAGCCCCTCTG
Caspase‐3	F: AACGGACCTGTGGACCTGAA
	R: TCAATACCGCAGTCCAGCTCT
GAPDH	F: GGCACAGTCAAGGCTGAGAAT
	R: ATGGTGGTGAAGACGCCAGTA
miR‐129‐1‐3p	F: AAGCCCTTACCCCAAAAAGAA
	R: CCAGTCTCAGGGTCCGAGGTATTC
U6	F: TGACACGCAAATTCGTGAAGCGTTC
	R: CCAGTCTCAGGGTCCGAGGTATTC

Abbreviations: F, forward; R, reverse.

**Table 3:** Sequences of primers used in PCR analysis of HL‐1 cells

	Sequences (5′‐3′)
GRIN2D	F: GGTGATGATGTTCGTCATGT
	R: TCCCAATGGTGAAGGTAGAG
Bax	F: TTTTGCTACAGGGTTTCATCCA
	R: GTGTCCACGTCAGCAATCATC
Bcl‐2	F: AGCCCACCGTAACAATCAAG
	R: CCTGTCCCTTTGTCTTCAGC
Caspase‐3	F: TCTGACTGGAAAGCCGAAACTCT
	R: AAAGGGACTGGATGAACCACGAC
GAPDH	F: CCTTCATTGACCTCAACTACATGG
	R: CTCGCTCCTGGAAGATGGTG
miR‐129‐1‐3p	F: GCCCTTACCCCAAAAAGTATAAA
	R: CCAGTCTCAGGGTCCGAGGTATTC
U6	F: GGTCGGGCAGGAAAGAGGGC
	R: GCTAATCTTCTCTGTATCGTTCC

Abbreviations: F, forward; R, reverse.

### 2.11 Western blot analysis

Cells were lysed in RIPA lysis buffer, and the protein concentrations were determined using the BCA method. The samples were subjected to SDS‐PAGE and transferred to PVDF membranes (Millipore). After blocking in 5% skim milk, the membranes were probed with primary antibodies (Table 4) overnight at 4°C, followed by anti‐rabbit secondary antibody (Table 4) for 2 hours at room temperature. The protein bands were visualized using enhanced chemiluminescence (ECL) (Beyotime) on a BioSpectrum Gel Imaging System (UVP). Relative protein expression was normalized to GAPDH.

**Table 4:** The antibodies used in Western blot analysis

Antibody	Dilutions	Source	Company
Primary antibodies			
GRIN2D	1:800	Rabbit	ABclonal, Wuhan, China
CALM1	1:800	Rabbit	ABclonal, Wuhan, China
CaMKⅡδ	1:800	Rabbit	ABclonal, Wuhan, China
SERCA2a	1:1000	Rabbit	Cell Signaling Technology (CST), USA
RyR2	1:800	Rabbit	ABclonal, Wuhan, China
RyR2‐pS2814	1:800	Rabbit	ABclonal, Wuhan, China
NCX1	1:1000	Rabbit	CST, USA
Bax	1:800	Rabbit	ABclonal, Wuhan, China
Bcl‐2	1:800	Rabbit	ABclonal, Wuhan, China
GAPDH	1:2000	Rabbit	ABclonal, Wuhan, China
Secondary antibody			
HRP Goat Anti‐Rabbit IgG	1:5000	Goat	ABclonal, Wuhan, China

### 2.12 Data analysis

All results are presented as mean ± standard deviation (SD). Data analysis was performed with the SPSS 19.0 and GraphPad 8.0 software. The Student's *t* test or one‐way ANOVA was applied to compare data from different groups. Statistical significance was defined as *P* < .05.

## 3 RESULTS

### 3.1 THP induces cardiomyocyte injury

**Figure 1:**
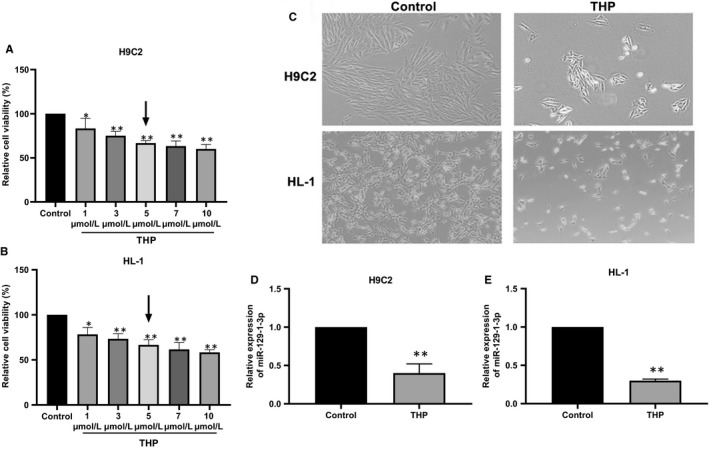
THP induces cardiomyocyte injury and down‐regulates miR‐129‐1‐3p. (A, B) H9C2 (A) and HL‐1 (B) cells were incubated with THP at indicated concentrations for 24 h. Cell viability was evaluated using the CCK‐8 assay. (C) H9C2 and HL‐1 cells were incubated with 5 μmol/L THP for 24 h. Representative microscopic images are shown. (D, E) H9C2 (D) and HL‐1 (E) cells were incubated with 5 μmol/L THP for 24 h. MiR‐129‐1‐3p transcript levels were determined using qRT‐PCR. n = 3, *P < .05 and **P < .01 vs Control

In accordance with reported THP cardiotoxicity, 24‐hour THP treatment dose‐dependently reduced H9C2 and HL‐1 cell viability as indicated in the CCK‐8 assay (Figure 1A,B). Microscopic examination revealed markedly decreased cell density along with changed cell morphology after 24‐hour incubation with 5 μmol/L THP (Figure 1C).

### 3.2 THP down‐regulates miR‐129‐1‐3p in cardiomyocytes

A recent miRNA microarray analysis performed in our laboratory revealed that miR‐129‐1‐3p was down‐regulated by THP in a rat model of THP‐induced myocardial injury.^24^ We further examined the effects of THP on miR‐129‐1‐3p expression in cardiomyocytes using qRT‐PCR. After 24‐hour treatment with 5 μmol/L THP, miR‐129‐1‐3p levels in H9C2 and HL‐1 cells were reduced to 41% and 32% of control, respectively (Figure 1D,E). Together, these in vitro and in vivo results implicate miR‐129‐1‐3p in the pathogenesis of THP‐induced cardiomyocyte injury.

### 3.3 MiR‐129‐1‐3p alleviates THP‐induced ROS production in cardiomyocytes

**Figure 2:**
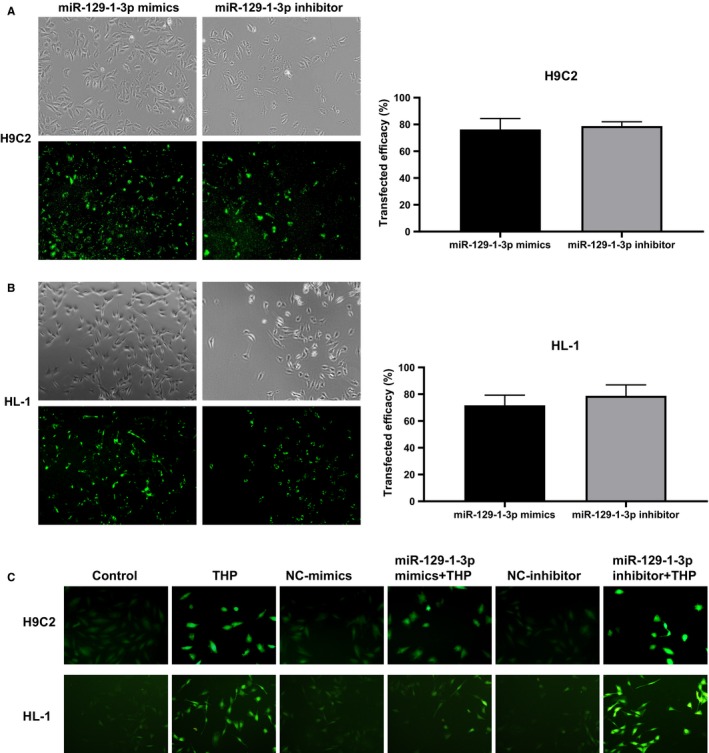
MiR‐129‐1‐3p alleviates THP‐induced ROS production in cardiomyocytes. (A, B) H9C2 (A) and HL‐1 (B) cells were transfected with the miR‐129‐1‐3p mimics or miR‐129‐1‐3p inhibitor for 8 h. Representative fluorescence images (×40 magnification, left) and quantified transfection efficiency (right, n = 3) are shown. (C) H9C2 and HL‐1 cells were transfected as indicated and treated with 5 μmol/L THP or vehicle alone for 24 h. Un‐transfected cells were included for comparison. Intracellular ROS levels were evaluated using the DCFH‐DA staining assay. Representative fluorescence images are shown (×100 magnification)

To investigate the functional role of miR‐129‐1‐3p in THP cardiotoxicity, we transfected H9C2 and HL‐1 cells with the miR‐129‐1‐3p mimics or miR‐129‐1‐3p inhibitor. As shown in Figure 2A,B, the miR‐129‐1‐3p mimics and inhibitor were successfully transfected into the cells with 70%‐80% transfection efficiency. Treatment with 5 μmol/L THP for 24 hours increased intracellular ROS levels in both H9C2 and HL‐1 cells, as indicated by the DCFH‐DA staining assay (Figure 2C). The ROS accumulation induced by THP was markedly attenuated by miR‐129‐1‐3p mimics transfection but aggravated by miR‐129‐1‐3p inhibitor transfection (Figure 2C). These data indicate that miR‐129‐1‐3p functions to mitigate THP‐induced oxidative stress in cardiomyocytes.

### 3.4 MiR‐129‐1‐3p protects cardiomyocytes from THP‐induced apoptosis

**Figure 3:**
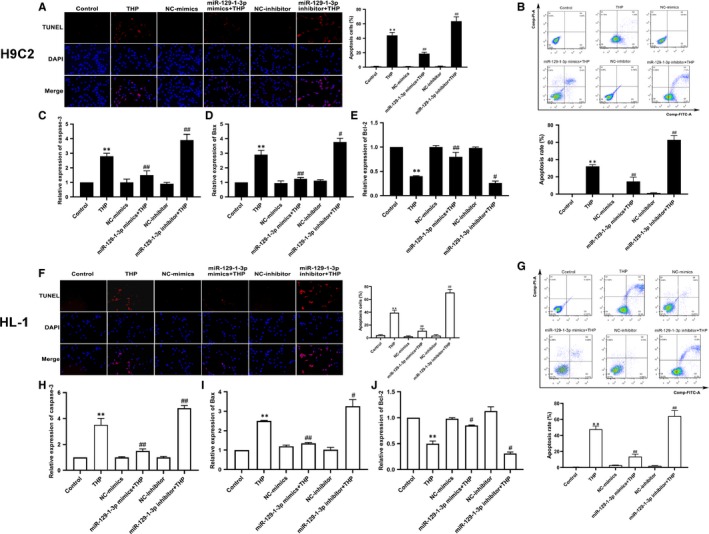
MiR‐129‐1‐3p protects cardiomyocytes from THP‐induced apoptosis. H9C2 and HL‐1 cells were transfected as indicated and treated with 5 μmol/L THP or vehicle alone for 24 h. Un‐transfected cells were included for comparison. (A, F) Cell apoptosis was evaluated using the TUNEL assay. Representative fluorescence images (×100 magnification, left) and quantified apoptosis rates (right) are shown. (B, G) Cell apoptosis was assessed using flow cytometry. Representative histograms (upper panel) and quantified apoptosis rates (lower panel) are shown. (C‐E) mRNA levels of Caspase‐3, Bax and Bcl‐2 in H9C2 cells were determined using qRT‐PCR. (H‐J) mRNA levels of Caspase‐3, Bax and Bcl‐2 in HL‐1cells were determined using qRT‐PCR. n = 3; **P < .01 vs Control; ^#^ *P* < .05 and ^##^ *P* < .01 vs THP

We next assessed apoptosis of H9C2 and HL‐1 cells using the TUNEL assay, as well as flow cytometry with Annexin V‐FITC/PI double staining. The mRNA levels of the apoptosis‐related proteins Caspase‐3, Bax and Bcl‐2 were determined using qRT‐PCR. TUNEL staining revealed drastically increased cell apoptosis after 24‐hour treatment with 5 μmol/L THP (Figure 3A,F). Flow cytometric analysis showed a higher percentage of total apoptotic cells, as well as a greater number of cells in early‐ and late‐stage apoptosis (Q2 + Q3) in the THP‐treated group compared with control (Figure 3B,G). The apoptosis‐inducing effects of THP observed in TUNEL staining and flow cytometry were supported by the qRT‐PCR analysis, which demonstrated that THP significantly up‐regulated Caspase‐3 and Bax, but down‐regulated Bcl‐2 (Figure 3C‐E,H‐J). These THP‐induced changes detected using TUNEL staining, flow cytometry and qRT‐PCR were alleviated by miR‐129‐1‐3p mimics transfection but exacerbated by miR‐129‐1‐3p inhibitor transfection (Figure 3A‐J), indicating that miR‐129‐1‐3p protects cardiomyocytes against THP‐induced apoptosis.

### 3.5 MiR‐129‐1‐3p is linked to the Ca^2+^ pathway by directly targeting GRIN2D

**Figure 4:**
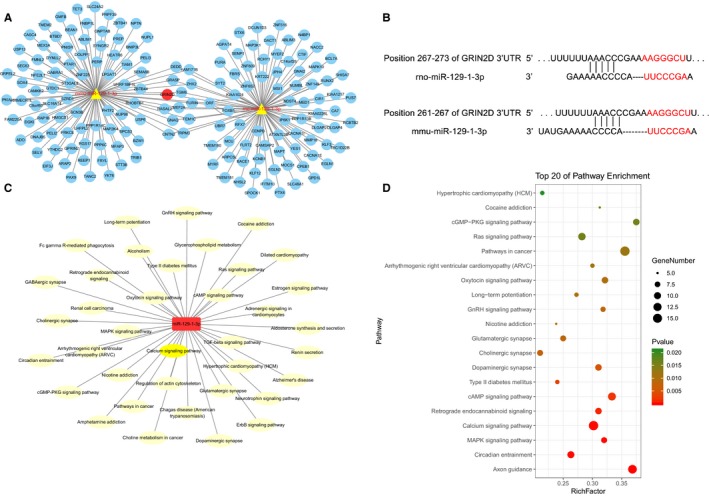
MiR‐129‐1‐3p is predicted to regulate the Ca^2+^ pathway by directly targeting GRIN2D. A, GO enrichment analysis of miR‐129‐1‐3p and its target genes. B, Diagram of tentative binding between miR‐129‐1‐3p and the 3′‐UTR of rat or mouse GRIN2D mRNA. C, KEGG pathway enrichment analysis of miR‐129‐1‐3p. D, The top 20 pathways dysregulated following THP‐induced myocardial injury

To uncover the molecular mechanisms underlying the protective effects of miR‐129‐1‐3p in cardiomyocytes, we performed GO enrichment analysis of miR‐129‐1‐3p and thereby revealed a potential link between miR‐129‐1‐3p and Ca^2+^ signalling (Figure 4A,C). Intriguingly, KEGG pathway enrichment analysis identified the Ca^2+^ pathway as one of the top 20 pathways dysregulated in THP‐induced myocardial injury (Figure 4D). Subsequent TargetScan database screening identified a potential miR‐129‐1‐3p‐binding site at the 3′‐UTR of GRIN2D mRNA (Figure 4B). Considering that GRIN2D is a subunit of the NMDA receptor Ca^2+^ channel, we speculated that miR‐129‐1‐3p regulates Ca^2+^ influx in THP‐treated cardiomyocytes by directly targeting GRIN2D.

**Figure 5:**
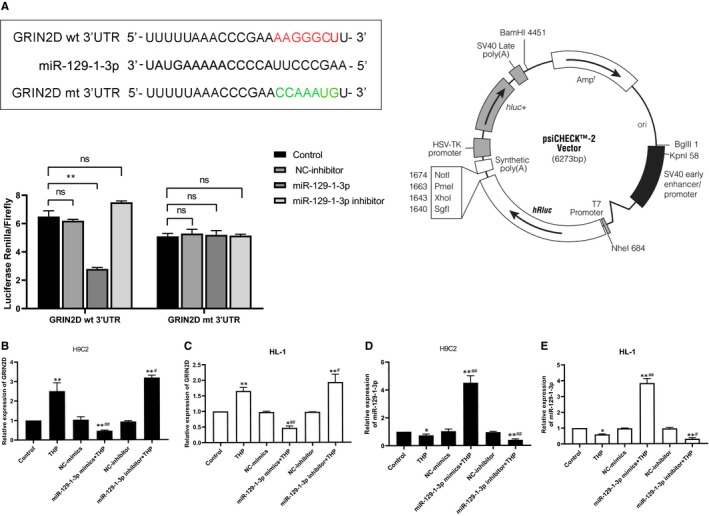
MiR‐129‐1‐3p directly targets GRIN2D. A, Diagram of the GRIN2D 3′‐UTRs containing the wild‐type or mutant miR‐129‐1‐3p‐binding site (left/upper), a diagram of the luciferase reporter vector (right) and the luciferase reporter assay data (left/lower) are shown. (B–E) H9C2 (B, D) and HL‐1 (C, E) cells were transfected as indicated and treated with 5 μmol/L THP or vehicle alone for 24 h. Un‐transfected cells were included for comparison. The levels of GRIN2D mRNA (B, C) and miR‐129‐1‐3p transcript (D, E) were determined by qRT‐PCR. n = 3; *P < .05 and **P < .01 vs Control; ^#^ *P* < .05 and ^##^ *P* < .01 vs THP

We subsequently confirmed that GRIN2D is a target of miR‐129‐1‐3p using a luciferase reporter assay (Figure 5A). Indeed, miRNA‐129‐1‐3p down‐regulation in response to THP challenge in H9C2 and HL‐1 cells was accompanied by increased GRIN2D expression (Figure 5B‐E). In addition, miRNA‐129‐1‐3p mimics transfection down‐regulated GRIN2D expression that was up‐regulated by THP, while miRNA‐129‐1‐3p inhibitor transfection had the opposite effect (Figure 5B,C). Together, these bioinformatics and cell‐based data strongly support that miRNA‐129‐1‐3p is linked to the Ca^2+^ pathway in cardiomyocytes by directly targeting GRIN2D.

### 3.6 MiR‐129‐1‐3p inhibits THP‐induced calcium overload in cardiomyocytes

**Figure 6:**
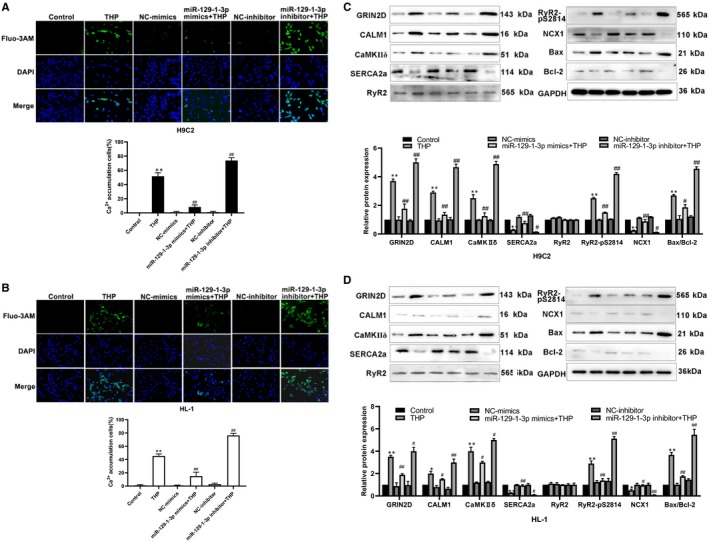
MiR‐129‐1‐3p inhibits THP‐induced calcium imbalance in cardiomyocytes. H9C2 (A, C) and HL‐1 (B, D) cells were transfected as indicated and treated with 5 μmol/L THP or vehicle alone for 24 h. Un‐transfected cells were included for comparison. (A, B) Intracellular Ca^2+^ levels were assessed using fluorescence imaging with Fluo‐3 AM staining. Representative fluorescence images (×100 magnification, upper panel) and percentages of Fluo‐3 AM‐positive cells (lower panel) are shown. (C, D) Protein expression of GRIN2D, CALM1, CaMKⅡδ, SERCA2a, RyR2, RyR2‐pS2814, NCX1, Bax and Bcl‐2 were determined using Western blot analysis. n = 3; *P < .05 and **P < .01 vs Control; ^#^ *P* < .05 and ^##^ *P* < .01 vs THP

To test the regulatory function of miR‐129‐1‐3p in Ca^2+^ signalling in cardiomyocytes, we assessed intracellular calcium levels using fluorescence microscopy combined with the Ca^2+^‐sensitive dye Fluo‐3 AM. As shown in Figure 6A,B, an increased number of Fluo‐3 AM‐positive H9C2 and HL‐1 cells was detected after 24‐hour treatment with 5 μmol/L THP, indicating THP‐induced calcium overload in the cells. The number of Fluo‐3 AM‐positive cells was significantly reduced by microRNA‐129‐1‐3p mimic transfection, but further increased by microRNA‐129‐1‐3p inhibitor transfection (Figure 6A,B), indicating that microRNA‐129‐1‐3p inhibits THP‐induced calcium overload in cardiomyocytes. Finally, we used Western blot analysis to evaluate the expression of proteins that are either key components of Ca^2+^ signalling or important regulators of intracellular Ca^2+^ trafficking/balance in cardiomyocytes including GRIN2D, calmodulin‐1 (CALM1), Ca^2+^/calmodulin‐dependent protein kinase IIδ (CaMKⅡδ), sarcoplasmic endothelial reticulum calcium ATPase 2 (SERCA2a), phosphorylated ryanodine receptor 2 (RyR2‐pS2814) and sodium calcium exchanger 1 (NCX1). As shown in Figure 6C,D, THP increased the expression of GRIN2D, CALM1, CaMKⅡδ and RyR2‐pS2814, but decreased the expression of SERCA2a and NCX1 in H9C2 and HL‐1 cardiomyocytes. The changes in expression of these proteins caused by THP were attenuated by miRNA‐129‐1‐3p mimics transfection but enhanced by miRNA‐129‐1‐3p inhibitor transfection. In support of our TUNEL assay and flow cytometry data on apoptosis, THP up‐regulated Caspase‐3 and the Bax/Bcl‐2 ratio, two markers of apoptosis, in H9C2 and HL‐1 cells (Figures 3 and 6C,D). MiRNA‐129‐1‐3p mimics transfection inhibited while miRNA‐129‐1‐3p inhibitor transfection enhanced the increases in Caspase‐3 and the Bax/Bcl‐2 ratio caused by THP (Figures 3 and 6C,D). Together, these results demonstrate that miR‐129‐1‐3p mitigates THP‐induced cardiomyocyte injury by inhibiting THP‐induced calcium overload and activation of Ca^2+^ signalling in cardiomyocytes.

## 4 DISCUSSION

Cardiovascular complications are common side effects of many anticancer therapies.^27^ This is attributed to the highly interconnected nature of the molecular pathways regulating tumorigenesis and cardiac function. For example, constitutive activation of signal transducer and activator of transcription 3 (STAT3) promotes breast cancer progression through regulating Bcl‐2, Bax, VEGF and MMP‐7.^28^ Meanwhile, STAT3 acts synergistically with myocardin to regulate Bcl‐2 and Bax in cardiomyocytes, consequently inhibiting cardiomyocyte apoptosis via the mitochondrial apoptotic pathway.^29^ Hence, efforts to target STAT3 in breast cancer had little success in the past because of the potential adverse effects, such as cardiotoxicity. THP, a new generation anthracycline antineoplastic drug, is frequently used to treat various solid tumours and haematologic malignancies.^30^ Although THP is more effective and less cardiotoxic than DOX,^5^ its cardiotoxicity still severely limits its clinical application. Various strategies have been exploited to control anthracycline cardiotoxicity. For example, liposome‐based formulations have been shown to greatly ameliorate the cumulative cardiotoxicity of THP.^31^ In addition, several drug classes, such as β‐blockers, statins, angiotensin‐converting enzyme inhibitors and dexrazoxane, have demonstrated beneficial effects in protecting against anthracycline‐induced cardiac damage.^32^

Despite decades of intense research, the pathophysiology associated with anthracycline cardiotoxicity is not fully understood. The leading hypothesis is related to iron‐mediated ROS production in cardiac tissues.^33^ Indeed, dexrazoxane, an EDTA derivative, is believed to protect against anthracycline cardiotoxicity by chelating iron and inhibiting iron‐anthracycline complex formation, consequently decreasing ROS regeneration.^34^ Disruption of mitochondrial calcium homoeostasis has also been implicated as a contributing mechanism for anthracycline‐induced cardiac injury.^35^, ^36^ Mitochondrial calcium overload can lead to mitochondrial permeability transition pore (mPTP) opening, further aggravating oxidative stress.^37^ In microscopic studies, Ca^2+^‐triggered mitochondria swelling was observed in cardiac tissues that suffered anthracycline‐induced damage.^38^ Additionally, calcium overload in cardiomyocytes may cause degradation of the myofilament protein titin, leading to sarcomere disruption and cell necrosis,^39^ further highlighting the role of calcium overload in the pathogenesis of anthracycline cardiotoxicity. However, the molecular mechanisms involved in anthracycline‐induced calcium overload in cardiomyocytes remain largely unknown.

In the present study, we found that miR129‐1‐3p, a potential biomarker of cardiovascular disease,^24^ is down‐regulated by THP in H9C2 and HL‐1 cardiomyocytes. Our GO and KEGG pathway enrichment analyses linked miR129‐1‐3p to the Ca^2+^ signalling pathway. Encouraged by these early results, we searched for potential targets of miR129‐1‐3p using TargetScan database screening. We identified a tentative miR129‐1‐3p‐binding site at the 3′‐UTR of GRIN2D, a subunit of the NMDA receptor calcium channel that is critical for calcium influx.^26^ By studying the effects of miR129‐1‐3p overexpression and knock‐down, we confirmed that miR129‐1‐3p directly regulates GRIN2D to ameliorate calcium overload and apoptosis of cardiomyocytes induced by THP challenge. These bioinformatics and cell‐based studies strongly suggest that miR129‐1‐3p plays a key role in THP‐induced cardiotoxicity through regulating calcium homoeostasis.

CALM1 and CaMK IIδ are key downstream effectors of Ca^2+^ signalling in cardiomyocytes.^40^, ^41^, ^42^, ^43^, ^44^, ^45^ Overactivation of Ca^2+^/CaMK IIδ signalling has been linked to cardiac muscle cell death, cardiomyopathy and heart failure.^45^, ^46^ In addition, SERCA2a, RyR2‐pS2814 and NCX1 are important regulators of Ca^2+^ homoeostasis in cardiomyocytes y.^47^, ^48^, ^49^, ^50^ In this study, we found that THP increased the expression of CALM1, CaMKIIδ and RyR2‐pS2814, but decreased the expression of SERCA2a and NCX1 in H9C2 and HL‐1 cardiomyocytes, and miR129‐1‐3p overexpression prevented the changes in the expression of these proteins induced by THP. Thus, miR129‐1‐3p inhibits THP‐induced calcium overload and imbalance as well as downstream Ca^2+^ signalling in cardiomyocytes by controlling the expression of GRIN2D, CALM1, CaMKIIδ, RyR2‐pS2814, SERCA2a and NCX1.

In summary, our data demonstrate that miR‐129‐1‐3p plays a central role in THP‐induced cardiotoxicity by inhibiting activation of the Ca^2+^ signalling pathway in cardiomyocytes. Our findings provide novel insights into the pathogenesis of THP‐induced cardiotoxicity and indicate that the miR‐129‐1‐3p/Ca^2+^ signalling pathway may serve as a new target for the development of novel cardioprotective agents to control anthracycline‐induced cardiac injury. Further studies to investigate this regulatory mechanism in appropriate animal models of anthracycline‐induced cardiotoxicity are warranted.

## CONFLICT OF INTEREST

The authors declare no conflicts of interest.

## AUTHOR CONTRIBUTIONS

QL conceived and designed the experiments. QL, MQ and TL performed the experiments. QL and QT analysed the data. ZG and PH contributed to the preparation of reagents/materials/analysis tools. QL wrote the manuscript. LR supervised this study.

## Data Availability

The data used to support the findings of this study are included within the article.
